# Importance of rumen ciliated protozoa and current research techniques for their analysis

**DOI:** 10.1099/mic.0.001786

**Published:** 2026-11-05

**Authors:** Sinéad Maloney, Neal T. Folliard, Gizem Karakaya, Sinéad M. Waters

**Affiliations:** 1 Teagasc, Animal and Bioscience Research Department, Animal and Grassland Research and Innovation Centre, Teagasc, Grange, Dunsany, Meath, Ireland; 2 School of Biological and Chemical Sciences and Ryan Institute, University of Galway, University Road, Galway, Ireland

**Keywords:** ciliate, microscopy, molecular approaches, protozoa, primers, rumen

## Abstract

Rumen protozoa are an integral component of the ruminant foregut microbiome, with a profound influence on feed digestion, microbial ecosystem dynamics and enteric methane emissions. Although they represent only a small proportion of species richness, protozoa contribute significantly to microbial biomass and functions in fibre degradation, volatile fatty acid production, microbial protein turnover, pH modulation and hydrogen-mediated methanogenesis. In addition to highlighting their importance to rumen function, this review summarizes classical and contemporary methodologies for enumerating, identifying and characterizing rumen protozoa. These methodologies include microscopy, fluorescent staining, flow cytometry and molecular approaches such as 18S rRNA, ITS and 28S rRNA amplicon sequencing. All major rRNA primers widely used over the last two decades are catalogued and their coverage is evaluated against current databases, highlighting the superior taxonomic resolution provided by ITS-based markers. The integration of single-cell sequencing and long-read platforms with multi-omics approaches (metagenomics, metatranscriptomics and metaproteomics) and advanced bioinformatic tools enables genome-resolved and functionally informed analyses of rumen protozoa. Methodological advances are integrated with functional insights to elucidate the contributions of protozoa to rumen ecology, feed efficiency and methane output. Key research gaps are also identified, including the need for improved microscopy techniques, automated image analysis, optimized cryopreservation protocols and robust culturing systems. This synthesis provides a comprehensive resource for researchers in microbial ecology, animal nutrition and environmental science, enabling precise protozoal profiling and informing strategies to enhance livestock productivity while mitigating environmental impacts.

**Impact Statement**

In rumen-based research, it is essential to study ciliated protozoa as they are a key component of the ruminant microbiome. This article provides a comprehensive overview of rumen protozoa, bringing together decades of research to highlight their importance in the rumen. It outlines how these micro-organisms influence digestion, microbial interactions and methanogenesis alongside their taxonomy at the genus level. Classical and modern methods for enumerating, identifying and functionally characterizing protozoa, including microscopy, fluorescent staining, flow cytometry and molecular techniques are discussed. Finally, areas that require future exploration are identified, including improvements in microscopy, the need for automated counting and image analysis for enumeration and identification, and enhanced culturing techniques. Addressing these issues will strengthen rumen microbiome research and increase our understanding of rumen protozoa. By integrating historical techniques, contemporary molecular approaches and functional insights, this review provides a comprehensive resource for the accurate profiling of protozoa. Understanding protozoal function has direct implications for the development of microbiome-based solutions for key issues including strategies to mitigate methane emissions and improve livestock productivity.

## Introduction

Protozoa are microscopic, single-celled eukaryotes with complex physiologies, several members of which are well adapted to inhabit anaerobic, moist environments [1–3] such as the carbohydrate-rich, microbially dense pre-gastric digestive systems of ruminant animals [4, 5]. Rumen protozoa were first described by Gruby and Delafond as far back as 1843 [6, 7]. They form distinct populations during early ruminant life with colonization evident within the first 2 weeks and mature communities visible within 80 days [4, 8–10]. There is evidence to suggest that initial colonization primarily occurs via calf-to-mother contact and progresses as the calf is introduced into the larger herd [2]. Community composition tends to remain relatively stable once established yet shifts may occur due to diet and environmental factors [8].

Whilst protozoa occupy a small proportion of species richness in the rumen microbiome, their large size means they contribute to the bulk of the microbial biomass (25–50%) [8]. A millilitre of rumen fluid can contain between 10^4^ and 10^6^ protozoa cells [11, 12]. To date, approximately 30 genera and 300 species of protozoa have been identified in the rumen microbiome [8]. The majority of rumen protozoa are ciliated; however, a small number of flagellated species are also present [13, 14]. Rumen ciliated protozoa are classified into two orders, based on differences in their morphology and metabolic functions: Vestibuliferida (commonly referred to as Holotricha) and Entodiniomorphida [1, 4]. Protozoa are primarily predators within the rumen ecosystem and contribute to other important functions such as fibre digestion, fermentation and microbial protein cycling [1, 7]. They depend on a symbiotic relationship with their host animal for plant material which they then convert into metabolites that the host can use [4]. Knowledge of their function in the rumen is somewhat restricted due to the challenges associated with maintaining axenic cultures of rumen protozoa when compared to their other rumen microbial counterparts [1, 2, 4]. However, recent advancements in omics technologies have increased the ability to study and understand the impacts of protozoa in the rumen ecosystem [1, 2]. This has significantly improved the information available on micro-organisms present in the rumen; however, knowledge is still limited regarding community interactions in these complex, multidomain ecosystems [2].

Providing *in vivo* context of rumen protozoa is challenging yet important for progressing microbiome-based technologies to improve livestock productivity and sustainability. This review aims to provide clear insights into the structures, functions and importance of protozoa species within the rumen ecosystem. It discusses key methodologies used in their study and identification, including classical microscopy techniques, genomic technologies and databases, and highlights important areas in which knowledge needs to be improved.

## Rumen protozoa community structure

### Key protozoa

Most rumen ciliated protozoa belong to the class Litostomatea within the Ciliophora phylum, with rumen ciliates confined mainly to two orders, Vestibuliferida and Entodiniomorphida [1, 15] (Fig. 1). Based on our current understanding, over 99% of the rumen protozoal community consists of 12 dominant genera [1]. The most prevalent genus is *Entodinium* across all ruminant types and species [1, 8]. Other abundant genera include *Isotricha, Polyplastron, Epidinium, Eudiplodinium, Diplodinium, Ophryoscolex* and *Dasytricha,* although abundance and community structure can vary depending on ruminant species and diet [1, 8, 12]. Within the rumen microbial community, the order Vestibuliferida contains two families, Isotrichidae and Paraisotrichidae, of large, uniformly ciliated protozoa [16]. The family Isotrichidae includes three genera: *Isotricha*, *Dasytricha* and *Oligoisotricha* and accounts for 40% of all rumen ciliates [15, 17]. The family Paraisotrichidae has one genus (*Paraisotricha*). Members of Isotrichidae can be identified by an ellipsoidal body; somatic ciliation is generally uniform (*Oligoisotricha* is an exception), and cells lack a concretion vacuole. There are three main Vestibuliferida species found in the rumen community, *Isotricha intestinalis, Isotricha prostoma* and *Dasytricha ruminantium* [4].

**Fig. 1.**
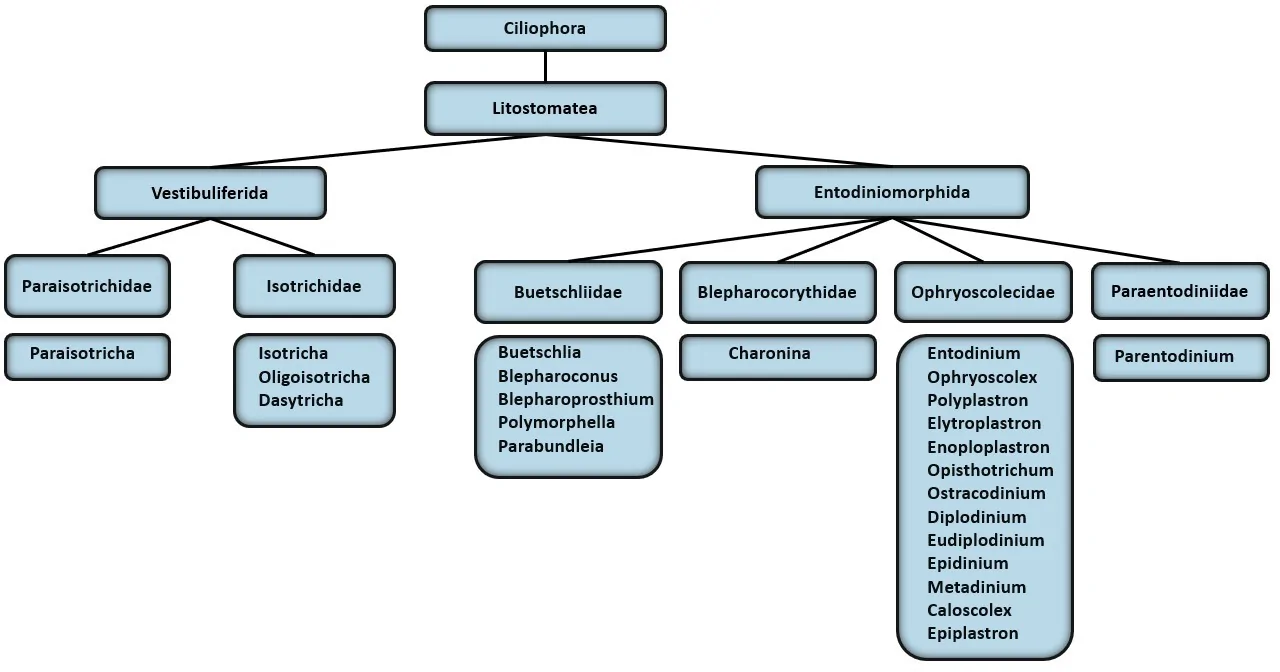
Taxonomic classification of rumen ciliated protozoa to the genus level.

The order Entodiniomorphida contains many families; however, only four are associated with the rumen environment: Ophryoscolecidae, Blepharocorythidae, Buetschliidae and Paraentodiniidae [16]. Members of this order are the most prominent protozoa within the rumen ecosystem. They are characterized by tufts of cilia at the polar ends of the cell, with extensive unciliated regions [18]. The Ophryoscolecidae family contains most of the rumen Entodiniomorphida [15]. Ophryoscolecidae are characterized by an ovoid body; cilia are limited to the anterior portion of the body (the adoral area), and in all genera, a left ciliary zone is present (except *Entodinium*). Ciliary zones are retractable, and all genera except *Entodinium* and *Diplodinium* have skeletal plates. The Ophryoscolecidae family is large, with 13 genera, including common *Entodinium*, *Diplodinium*, *Eudiplodinium*, *Ostracodinium* and *Metadinium*. The main genera in the rumen are typically *Entodinium* and *Diplodinium*, although it has been found that community composition varies between ruminant species [1, 15, 19]. For Blepharocorythidae, somatic ciliation is reduced. They possess ciliary zones at the anterior and posterior ends of the cell and have a distinct vestibulum provided with cilia throughout its length. The genus *Charonina* is present in this family. The Paraentodiniidae family has one genus *Parentodinium*.

### Rumen community structures

Rumen protozoa communities can be categorized into one of four community types (Types A, B, O and K), characterized by species composition [20, 21]. These community structures were initially described using inoculation and microscopy studies, in which young ruminants were inoculated with mixed cultures of protozoa and community composition was assessed microscopically in rumen fluid samples [22]. The four community types are influenced by substrate preference, host specificity and antagonistic interactions between protozoal species, with community stability dependent on host factors [20, 23]. The most common protozoa genera found in the rumen community, *Entodinium, Dasytricha and Isotricha*, are shared across all community types, which differ based on the presence of less dominant species [21, 24]. Type A is defined by the presence of *Polyplastron multivesiculatum,* which is often associated with *Diploplastron affine* and *Ophryoscolex* [21, 22, 25]. Type B is defined by the presence of *Epidinium* and *Eudiplodinium*, individually or together, and is enriched with *Diplodinium* [2, 22, 25]. Type O is exclusively composed of *Entodinium, Dasytricha and Isotricha*, and Type K contains only *Elytroplastron bubali* [20–22]. Types A and B are the most common of the four community structures, with Type A most frequently observed in sheep and Type B most associated with cattle [22]. Type K is also specifically found in cattle [20, 21]. Certain protozoa species do not establish mixed communities due to antagonistic interactions, making these community types distinct [22]. Inoculating young ruminants that have an established Type B community with Type A inocula results in the rapid elimination of the Type B community, with *P. multivesiculatum* becoming dominant within 48 h [22]. *Polyplastron* is highly predatory and will eliminate *Epidinium* and *Eudiplodinium* if it becomes established in the microbial population, resulting in Type A community structures dominating over Type B due to predation [26]. It was also observed in flocks of sheep that approximately equal numbers of sheep within the flock will have Type A community as Type B community and pen mates share similar community structures [9, 21]. These findings suggest that an unknown host-specific factor influences the colonization of protozoa in the rumen [9]. Reasons for observed differences in protozoa populations between individuals remain unknown, but it has been postulated that host genomics and protozoa predation may be possible causes [15]. The findings in preliminary inoculation and microscopy studies have since been confirmed by DNA-based techniques, including phylogenetic marker genetics and subsequent RNA and protein analysis [2, 22]. Studies have shown few functional differences between the communities, but abundances of specific bacterial, archaeal and protozoal populations diverge between them [2, 9]. There were also few effects on animal performance and microbial metabolism, which underlines the plasticity and functional redundancy of the rumen microbial community [2]*.*

## Functions of rumen protozoa

The presence and distribution of protozoa in the rumen have been well documented since their discovery by Gruby and Delafond in 1843. Despite this, knowledge of the genomic and functional roles of rumen protozoa remains limited [27]. Early taxonomic, morphological and metabolic studies relied mainly on classical techniques, including culture-based experiments, microscopy and defaunation studies [4, 28]. It was demonstrated that rumen protozoa contribute to carbohydrate fermentation and predation and interact with other members of the rumen microbial community [28]. However, these contributions were mostly implied by these early studies due to difficulties associated with attaining protozoa cultures for accurately identifying species under the microscope and the maintenance of defaunated ruminants [5, 29]. Therefore, species-specific functions remain poorly characterized, and many rumen microbial studies have excluded protozoal influence on overall rumen function [27, 28].

Discernment of protozoal function is difficult using culture-based methods as protozoa are extremely difficult to culture axenically [27]. Not only is it difficult to replicate their exact environmental and nutritional requirements, but protozoa cannot grow in the absence of live bacteria, which provide protozoa with most of their nitrogen requirements [4, 15]. Furthermore, bacterial presence or contamination of protozoa cultures makes it difficult to recognize specific contributions of protozoa to certain functions and assign characteristics to individual protozoa species [4]. There is still no significant progress in achieving axenic cultures of rumen protozoa to allow for accurate culture-based metabolic studies.

Protozoa are not essential to the survival of ruminant animals and their removal from the rumen microbiome is possible [8]. This is called defaunation and allows for controlled *in vivo* experiments to assess the role of protozoa in the rumen environment. The entire protozoal community or individual subpopulations are added or removed from the rumen microbiome and the resulting effects can be observed [28]. Interpretation of defaunation studies is challenging, as it is difficult to distinguish between the direct effect of protozoa or residual changes to the microbial community induced by defaunation [7, 30]. There are no commercially viable defaunation techniques currently available and it remains unclear what the long-term effects of defaunation are on animal health and productivity [7, 31]. Defaunated animals are also difficult to maintain and are largely impractical in commercial settings [1].

The limitations of classical methods to study rumen protozoa have been eased by the development and expansion of genomic technologies. Recent omics studies and the publication of the total genome sequence of *Entodinium caudatum* have provided more detailed insight into genetic information of rumen protozoa and their metabolic activity [5, 30, 32]. However, they remain significantly understudied compared to other members of the rumen microbiome and metagenomic research on rumen protozoa continues to be challenging due to the complexity of the protozoal genome [27, 28]. Rumen protozoa have macro- and micronuclei, each possessing its own unique genome [5, 29]. Ciliate protozoa are very metabolically active in the rumen, influencing fermentation pathways, the proportion of fermentation end-products and the composition and abundance of the microbial population [18]. A considerable share of the overall rumen microbial metaproteome belongs to *Epidinium, Entodinium and Isotricha* species, emphasizing protozoa as key contributors to the rumen environment [27]. Further advances in omics-technology allow for single-cell sequencing and genome assembly to generate high-quality rumen ciliate genomes and comparative genomic analysis to identify enzymatic sequences associated with key metabolic functions in the rumen [30, 33]. The expression of certain enzymes indicates that protozoa contribute to carbohydrate metabolism, host–microbe symbiosis, predation and protein cycling within the rumen [1, 5, 30]. Noticeable differences between the enzymatic profiles of Vestibuliferida and Entodiniomorphida highlight the divergent functions and different metabolic niches of these two groups within the rumen environment [29]. A recent study generated 450 medium-quality ciliate genomes, 391 of which were newly sequenced, representing a significant breakthrough in rumen protozoa research, establishing a framework for rumen protozoa genomics [33]. This work unveiled a new hydrogen-producing organelle, strengthening the links between protozoa and methanogenesis in the rumen [33]. There is also evidence that protozoa participate in pH buffering and volatile fatty acid (VFA) production [1, 5].

### Carbohydrate metabolism

Protozoa exhibit a rich and diverse metabolic profile to facilitate polysaccharide decomposition and enhance host digestion [34, 35]. Their role in plant biomass degradation was initially suggested by microscopy, with protozoa observed to closely associate with plant material and actively participate in its breakdown [36, 37]. Enzyme activity assays from *in vitro* cultures or protozoa isolated from rumen contents revealed specific enzymatic activities including cellulolytic, hemicellulolytic and glycoside hydrolase activity [38–41]. These enzymes belong to a group known as carbohydrate active enzymes (CAZymes). They are a diverse set of enzymes involved in carbohydrate synthesis, degradation and modification [42]. They exhibit high substrate specificity and vastly enhance carbohydrate metabolism [43]. There are five main CAZymes, all of which are present in rumen protozoa [34]: glycoside hydrolases (GH), polysaccharide lyases, glycosyltransferases, carbohydrate esterases and auxiliary enzymes [42]. It is reported that protozoa acquired their suite of CAZymes via horizontal gene transfer (HGT) from bacteria [30, 44]. Fibrolytic activity had also been inferred from defaunation and *in vitro* studies, with defaunated ruminants showing reduced fibre digestion and a 7% reduction in organic matter digestibility compared with fully faunated counterparts [1, 10]. Protozoa can also indirectly improve fibre digestion through their association with fibre-degrading bacteria. Despite being anaerobic, protozoa scavenge free oxygen that may enter the rumen, helping to maintain an anaerobic environment, which is crucial for fibrolytic bacteria [1, 45].

Biotechnology has enhanced these preliminary methodologies, providing further evidence for the protozoal contribution to plant degradation in the rumen [35]. A meta-omics study found that 8% of protozoal metatranscriptomic reads were involved in carbohydrate metabolism and concluded that the carbohydrate metabolism represents a major role of rumen protozoa [29]. The development of meta-omics and sequencing technologies allows for screening of specific enzymes in rumen protozoa [46]. Molecular cloning and bioinformatics have directly identified genes encoding cellulolytic and hemicellulolytic enzymes, and the sequencing of the *En. caudatum* genome identified several CAZymes associated with starch and hemicellulose degradation [32, 47, 48]. CAZyme analysis revealed significant metabolic diversity and substrate specificity of protozoa taxonomic groups [34]. Single-cell transcriptomics revealed an extensive repertoire of CAZymes and other lignocellulases in individual protozoa species, consistent with studies that identified these genes in mixed ciliate populations [35]. Additionally, CAZyme activity has been detected at higher frequencies in protozoa proteomes than in bacterial proteomes, signifying protozoa as key contributors to carbohydrate digestion in the rumen [27].

Some protozoa species encode synergistic enzymes capable of degrading polysaccharides into oligosaccharides and breaking them down completely [30]. Functional genomic analysis reveals differences in the metabolic potential of individual rumen ciliate taxa. For example, Cedrola *et al*. [34] identified 4,243 CAZymes across 33 ciliate genomes. The diversity of CAZyme profiles among taxa suggests functional division and niche separation.

Entodiniomorphida and Vestibuliferida differ in their substrate preference [17]. Entodiniomorphida species tend to target fibrous plant material, whereas Vestibuliferida prefer sugars and non-structural carbohydrates [17]. Substrate specialization is also present within subfamilies of the rumen ciliate population. In the Ophryoscolecidae family (Entodiniomorphida), Entodiniinae utilize non-structural carbohydrates, whereas Diplodiniinae and Ophryoscolecinae exhibit a preference towards structural carbohydrates [30, 34]. The same is observed in Isotrichidae (Vestibuliferida) where *Isotricha* species degrade non-structural polysaccharides but *Dasytricha* specializes in fibre degradation [34].

The sequencing of *En. caudatum* revealed the presence of lignocellulases and GHs, which are key fibre-digesting enzymes [1]. *Epidinium* spp. (Entodiniomorphida) are among the most fibrolytic rumen ciliates, evidenced by the vast expression of CAZymes [2]. Furthermore, these species are reported to be most closely associated with plant material and actively forage for plant chloroplasts [2, 15]. Often in rumen environments where *Epidinium* spp. are highly prevalent and active, their activity and abundance have an impact on the availability of carbohydrates for other rumen micro-organisms [2].

### Microbial protein cycling and predation

Microbial protein is a key protein source for ruminant animals, contributing over 50% of amino acid and crude protein requirements [49, 50]. Rumen protozoa provide up to 30% of the microbial protein supply to ruminants due to their large biomass and high digestibility [7]. They also influence intraruminal nitrogen recycling, as evidenced by increased bacterial abundance, increased protein flow to the small intestine, decreased rumen ammonia concentration and improved nitrogen use efficiency in defaunated animals [1, 7, 51]. However, protozoa are also considered damaging to nitrogen metabolism in the rumen due to their predatory behaviour [52]. Furthermore, 60–80% of protozoa lyse or die in the rumen, requiring their degradation so that nitrogen can be recycled [53]. Rumen protozoa cannot synthesize their own amino acids and rely on preformed amino acids for protein synthesis [54]. They use digestive enzymes to engulf and digest microbial cells and convert microbial proteins into peptides, amino acids and ammonia, decreasing the availability of microbial protein, therefore, decreasing ruminal nitrogen use efficiency [1, 52, 55]. The majority of protozoal nitrogen requirements are acquired through bacterial predation with the remaining obtained from free amino acids and peptides in the rumen fluid [51]. However, rumen ciliates are highly inefficient at utilizing nitrogen and much of the peptides and amino acids are converted to urea and ammonia, which cannot be used by protozoa and are subsequently released back into the rumen fluid [1, 13, 51]. This is a nutritionally wasteful process and decreases protein supply to the ruminant host [52]. Rumen protozoa are primary predators within the rumen microbial community, and it has been suggested that protozoa can engulf up to 24% of the rumen bacteria population per day [27, 55, 56].

Proteolytic activity in rumen protozoa was initially detected using enzymatic assays and defaunation studies with evidence of proteinase activity observed in both mixed and single species ciliate populations [57, 58]. The proteolytic characteristics between genera appear similar; however, gel electrophoresis detected multiple forms of proteinases, suggesting interspecies differences within both Vestibuliferida and Entodiniomorphida [57]. Defaunation studies revealed differences in bacterial communities between faunated and defaunated animals; however, it was unclear whether this was due to predation, symbiotic relationships or other indirect effects of defaunation [59]. Defaunated ruminants appeared to have higher rumen nitrogen use efficiency, and it was suggested that this resulted from a lower rate of predation [59]. A positive correlation between the abundance of Entodiniomorphida and the rate of bacterial degradation implies that the Entodiniomorphida genera play a critical role in bacterial protein degradation [60]. It was also indicated that protozoal cell size directly relates to the rate of bacterial degradation, with bigger protozoa having higher degradative activity [60]. Experimental evidence suggests that *En. caudatum* is the most predatory species of the characterized rumen ciliates [61]. Multi-omics studies detected phagosomes, lysozymes, chitinases and enzymes involved in endocytosis supporting experimental evidence of predatory behaviour [1, 8]. The expression of peptidases also indicates degradation of engulfed microbial proteins [62]. Fungal predation by rumen ciliates was detected in early microscopy studies of protozoa observing the engulfment of fungal zoospores, and it was discovered that fungal numbers are reduced by protozoa presence *in vivo* [63, 64]. This is further supported by the expression of chitinases to degrade the chitin-rich fungal cell walls [17, 29].

### Contribution to rumen fermentation

Protozoa contribute significantly to rumen fermentation through their involvement in carbohydrate metabolism, thereby influencing VFA and assisting in rumen pH stabilization. VFA are produced as a by-product of carbohydrate fermentation and account for 70–80% of the ruminant metabolic energy requirements [13]. The main VFA produced in the rumen are acetate, propionate and butyrate at a typical ratio of 75:15:10, respectively [65]. Livestock feed is often rich in fermentable carbohydrates, which are readily available to micro-organisms for fermentation. If fermentation occurs too quickly after feeding, this can result in an accumulation of VFA in the rumen, subsequently decreasing rumen pH [66]. Prolonged or extensive reductions in rumen pH can cause rumen acidosis posing a serious health risk to the animal [66]. Protozoa rapidly engulf starch and sugar molecules in the rumen, sequestering them from bacterial fermentation [7, 67]. Vestibuliferida species have been observed to assimilate sugars and convert them to amylopectin, whereas Entodiniomorphida species are associated with engulfing starch and storing it as glycogen [67, 68]. The stored carbohydrates are released and fermented in a more controlled manner, stabilizing VFA production, preventing excessive accumulation. This reduces the magnitude of the post-feeding drop in pH helping to maintain rumen pH [67, 68]. This function is especially important in intensive livestock production systems, which often use high quantities of starch-rich feed, increasing the risk of rumen acidosis [66, 69]. The role of protozoa in maintaining rumen pH is crucial for rumen homeostasis as rumen micro-organisms are sensitive to incorrect pH. Rumen ciliates were observed to be eliminated when pH dropped below 5.5, indicating that protozoa themselves are vulnerable to acidic conditions [70]. This is supported by reports of higher rumen fluid pH in normal protozoa populations compared to defaunated ruminants [68].

Genomic studies on *En. caudatum* uncovered pathways connected with acetate and butyrate production [5]. This was supported by multi-omics studies, which found enzymes involved in acetate and butyrate production are present in *Entodinium* and *Epidinium* providing further evidence for the contribution of protozoa to VFA production [27]. Despite this, difficulties in culturing protozoa have made it challenging to verify specific VFA produced by individual protozoa species [1, 27]. Variations in rumen protozoa abundance are linked to differences in VFA production and composition, as demonstrated by defaunation studies [7, 71]. While there is still no definitive evidence of specific VFA produced by individual protozoa, the substrate preferences of Entodiniomorphida (starch) and Vestibuliferida (sugars) indicate that the former is mainly associated with acetate production and the latter linked to butyrate production.

### Rumen protozoa and methanogenesis

Methanogenesis is vital to remove excess hydrogen produced during enteric fermentation and is the main hydrogen sink in the rumen [8, 72]. Similar to the accumulation of VFA, a build-up of hydrogen in the rumen decreases rumen pH, increasing the risk of rumen acidosis [73]. Rumen protozoa are an important source of hydrogen and, coupled with their ability to scavenge oxygen, create favourable conditions for methanogenesis [19]. Methane production associated with ciliated protozoa is largely attributed to symbiotic relationships with rumen methanogens [74]. Numerous studies using FISH and electron microscopy demonstrated that rumen protozoa form both ectosymbiotic and endosymbiotic associations with the methanogenic archaea [75–78]. These associations are formed using methanogen–protozoa-specific adhesin-like proteins, and it is estimated that 9–37% of enteric methane is produced by methanogens physically associated with protozoa [8, 27, 79]. One single ciliate protozoon can harbour up to 10^4^ methanogens [56]. *Methanobrevibacter (Mbb*) spp. responsible for the main methanogenic pathway in the rumen are also reported as the predominant protozoa-associated methanogens [79, 80]. Symbiotic relationships have been reported in several common protozoa genera, including *Entodinium*, *Polyplastron*, *Epidinium* and *Ophryoscolex,* generally involving members of the orders Methanobacteriales and Methanomicrobiales among the methanogenic archaea [31]. Intracellular methanogens are found within many rumen protozoa, whereas extracellular methanogens are less prevalent [75]. Differences have been observed in the endosymbiotic methanogens associated with Vestibuliferida and Entodiniomorphida [79]. Inoculation of *Polyplastron multivesiculatum* (Entodiniomorphida) into the rumen was associated with the detection of *Methanobrevibacter bryantii, Methanobrevibacter ruminantium* and *Methanosphaera stadtmaniae* [56]. Contrastingly, *Isotrichidae* spp. (Vestibuliferida) were often associated with *Methanobrevibacter smithii* [56]. These findings suggest that dominance of certain protozoa can influence the composition of the methanogen community and may allow for more targeted defaunation approaches to reduce methane emissions. However, the difficulty in achieving protozoa cultures once again makes it challenging to prove specificity of these relationships [56]. It is also suggested that ectosymbiotic relationships predominantly occur in members of Entodiniomorphida, with very little evidence of these relationships observed in Vestibuliferida, although this difference does not appear to affect methane production [75].

It is well established that protozoa occupy a key role in stimulating methanogenesis in the rumen, yet the molecular mechanisms that they employ remain poorly understood [7, 33, 81]. Their role in methanogenesis has been emphasized by defaunation studies, which observed up to 35% reductions in methane yield [8, 19, 82]. However, a lack of high-quality reference genomes and taxonomic frameworks have yielded inconsistent conclusions regarding how exactly rumen ciliates impact methanogenesis [33]. Positive linear correlations have consistently been observed between rumen protozoal abundance and metabolic activity and methane emissions [1, 8, 83]. This was highlighted recently by Xie *et al.* [33], who sequenced 450 ciliate genomes, analysed rumen metagenomic and metatranscriptomic datasets, and measured *in vivo* methane emissions from a cohort of dairy cows. It was observed that most genera within both Vestibuliferida and Entodiniomorphida have a positive correlation with methane yield and methanogen abundance [33]. It is also reported that greater protozoa abundance is associated with higher abundance of the dominant rumen methanogens *Methanobrevibacter* and *Methanomicrobium* [84]. These findings from Xie *et al.* [33] confirm a robust relationship between rumen protozoa and methanogens, which are further supported by *in vivo* studies, emphasizing the role of protozoa in rumen methane production [45]. It is thought that protozoa–methanogen symbiotic relationships related to high ruminant methane emissions are driven by interspecies hydrogen transfer [27]. The large quantities of hydrogen produced by protozoa increase hydrogen availability for methanogens, preventing its redirection to alternative hydrogen sinks, allowing for methanogenesis [8, 79]. A single protozoa cell can produce between 0.5–5 nmol of hydrogen per day [56, 75]. Removal of hydrogen by methanogenesis benefits protozoa, as excessive hydrogen accumulation can inhibit protozoal metabolic activity [79]. It has long been hypothesized that rumen ciliates generate large quantities of hydrogen through hydrogenosomes, similar to other anaerobic protozoa, thereby facilitating interspecies hydrogen transfer and promoting methanogenesis [8, 33, 79, 81]. While many rumen protozoa do contain hydrogenosomes, other dominant species within the rumen environment such as *Entodinium* lack these organelles [85]. However, a recent study discovered a new hydrogen-producing organelle, the ‘Hydrogenobody’, which connects rumen ciliate activity with methane emissions and explains taxon-specific influences of ciliates on methane production [33]. These newly discovered organelles are distinct from hydrogenosomes in structure, cellular location and how they produce hydrogen. By constructing a novel rumen ciliate genome catalogue, it was discovered that rumen protozoa express unique hydrogenase enzymes [33]. Originally, it was thought that the hydrogenase enzyme entered hydrogenosomes; however, this study revealed that rumen protozoa hydrogenases lack a mitochondrial or hydrogenosomal target sequence, which is indicative of hydrogenosome activity. The absence of this target sequence, but the presence of a signal peptide, indicates that the enzyme instead enters into a secretory pathway. Using immunofluorescence and electron microscopy, it was observed that hydrogenase activity clustered in oval-shaped vesicles around the basal body of cilia [33]. This is contrary to previous assumptions that hydrogenase activity occurs in cytoplasmic hydrogenosomes [86, 87]. The hydrogenobodies were detected across a diverse range of rumen ciliates, including the *Entodinium* species, which were previously believed to lack hydrogenosomes [85].

The discovery of the hydrogenobody also identified the location of oxygen consumption by rumen protozoa [33]. Flavodiiron proteins act as oxygen reductase enzymes in rumen ciliates and their activity was observed in a similar pattern to hydrogenase, suggesting that these enzymes are also located in the same location [33]. The ability of protozoa to scavenge for oxygen was previously assumed to maintain anaerobic conditions to benefit methanogens [19]. These new findings also imply that flavodiiron proteins maintain an anaerobic microenvironment within hydrogenobodies to protect oxygen-sensitive enzymes such as hydrogenase.

Differences in metabolic activities between Vestibuliferida and Entodiniomorphida and among the Entodiniomorphida subfamilies indicate that some protozoa have a greater impact on methanogenesis than others [79]. A study found strong positive correlations between total rumen protozoa abundance and methane emissions, driven by Vestibuliferida species, but no such correlation was observed for the Entodiniomorphida population [19]. This is supported in the study by Xie *et al*. [33] concluding that differences in the methane promoting potential arise from variations in hydrogenobody abundance. Since hydrogenobodies are localized at the basal bodies of cilia, there is a direct positive correlation between hydrogenobody abundance and cilia coverage [33]. Vestibuliferida have greater ciliate coverage than Entodiniomorphida species and hence possess a higher abundance of hydrogenobodies, visually confirmed by electron microscopy [33, 88]. It has long been reported that Vestibuliferida species are more influential on methanogenesis than Entodiniomorphida due to greater hydrogen production and oxygen consumption [8, 19, 79]. The discovery of the hydrogenobody supports this, although it is now known that these activities occur in the same organelle and not in the hydrogenosomes, and taxonomic influence is directly related to cilia coverage [33]. Therefore, investigating the differences in methanogen–protozoa relationships at the species level may provide clearer insight into species-specific influence on methanogenesis compared with assessing the entire protozoal community. For example, the role of Vestibuliferida in methanogenesis is supported by studies in which defaunated sheep were inoculated with *Isotricha* spp., resulting in similar methane emissions to fully faunated sheep [79]. However, it was unclear whether this resulted from the presence of Vestibuliferida-associated methanogens generally or from a specific methanogen species [79]. Furthermore, *in vitro* fermentation studies indicate that the presence of *Dasytricha* species amplifies abundance and metabolic activity of methanogens and promotes methane production [33]. This is reflected by *in vivo* studies, which found *Dasytricha* to be 100-fold higher in high methane emitting sheep and also related to an increased abundance of methanogens in comparison to low methane emitting sheep [89].

### Rumen protozoa as targets for methane mitigation

Rumen protozoa clearly have a significant contribution to methanogenesis in ruminants and may account for up to 47% of the variation in methane production [75]. Rumen protozoa could represent a potential target for enteric methane mitigation as defaunation has been observed to reduce methane emissions by an average of 11% with some studies achieving reductions of up to 35% [8, 19, 82]. However, the cause of methane reductions attained by defaunation remains elusive. It is suggested that decreased methanogenesis results from a decline in the methanogen population, but these correlations are only moderate [79]. Defaunation may also decrease hydrogen supply to methanogens, thereby inhibiting methanogenesis, although some studies report that other rumen microbes can fill this niche [75]. Additionally, defaunation is linked with reduced plant material degradation in the rumen, which may account for the decrease in methane emissions [75, 90]. *In vitro* defaunation studies found that decreased feed digestion did not reduce methane production, contrary to *in vivo* findings [82, 91]. Other studies suggest that the impact of defaunation on methane emissions may be diet dependent [31]. Findings indicate that high-grain diets had a greater reduction in methane emissions than high-forage diets. It was suggested that the absence of protozoa from the rumen removed the main hydrogen source for methanogenesis in the high-grain diets [92]. The long-term effects of defaunation are elusive as achieving total removal of protozoa is technically difficult and challenging to maintain [1, 31]. It is also an impractical methane mitigation strategy in animal production systems.

The influential divergence in methanogenesis between Vestibuliferida and Entodiniomorphida, emphasized by the discovery of the hydrogenobody, highlights the potential of targeting Vestibuliferida for methane reduction [33]. Skatole and indole compounds were discovered to inhibit Vestibuliferida protozoa species and could be adapted to specifically target Vestibuliferida species within the rumen microbial community [93, 94]. Improved bacterivory strategies targeting Vestibuliferida-specific hydrogenobodies and their enzymes could offer targeted molecular methane mitigation [33]. It is hypothesized that targeting Vestibuliferida species specifically will minimally impact fermentation and overall rumen function as Entodiniomorphida species are more dominant in the carbohydrate metabolism and microbial community structure [33].

## Role of microscopy in rumen protozoa studies

It is over 180 years since protozoa were first visualized under a light microscope, and the study of these micro-organisms began [6]. Rumen ciliate protozoa vary in size from ~18 to 500 µm and can be recognized at relatively low magnification [95]. Complex physiological features, including mouth-like adoral openings and a tongue-like extrusible peristome, which helps ingest feed particles into an oesophagus-like structure, can be observed [96]. For decades, microscopy-based techniques and manual counting have been used as the gold standard to enumerate and differentiate between ciliate species in rumen studies based on cell morphology [97]. However, counting ciliates is slow and laborious due to their polymorphic nature, and differentiation to the genus or species level requires a high level of expertise [98]. Despite this, for many years, microscopic enumeration has had several advantages over PCR-based molecular methods [7]. Firstly, until recently, there was a lack of 18S rRNA gene sequences for many observed genera and species [7]. Secondly, copy number variation of rRNA genes across different genera or under different growth conditions may skew the observed proportions of genera [99]. Using 18S rRNA techniques, small protozoa such as *Entodinium* tend to be underestimated, and much larger protozoa such as *Epidinium* or *Polyplastron* tend to be overestimated when compared to microscopic techniques [100]. These discrepancies are largely attributed to incomplete databases and rRNA gene copy number variation in comparison to using microscopy. In an attempt to reduce labour, a recent study looked at incorporating automated image-based technology into the counting procedure using the YOLACT algorithm combined with staining (using brilliant green, methylene blue and Lugol’s iodine). Authors reported promising results in identifying species, with an average accuracy of 89.5% [101].

### Microscopy counting techniques

The study of rumen ciliates using microscopic techniques is not only important in live animal studies but also when using *in vitro* techniques, where survival rates of ciliates can be low [102–104]. This is especially important in *in vitro* dietary studies to ensure diets are assessed with the full complement of the rumen microbiome. When collecting samples for protozoal study, care is needed when harvesting rumen digesta by transoesophageal collection, rumen fistulation or from slaughtered cattle, and when separating rumen fluid from whole and mixed rumen contents, as these procedures can affect the distribution of genera within the sample [95]. Filtering digesta through cheesecloth is often used to remove plant debris; however, care is needed when selecting the number of layers to use, as cheesecloth with a pore size of 50 µm or less could substantially reduce the number of large protozoa present [105].

The most common protozoa counting procedure is the preservation of rumen fluid samples by fixation in 50% formalin (18.5% formaldehyde) [78, 100, 101]. A stain is then added to aid visualization under the microscope and distinguish between species (Fig. 2). There are many options of stains for highlighting certain anatomical structures within the protozoa. For total protozoal cell counts, visualization of the entire cytoplasmic membrane is required and brilliant green dye is often used as it provides uniform staining of the entire cell [100, 101]. A Sedgwick–Rafter counting chamber, which is divided into 1,000 grids, is used for the counting procedure as this provides a fixed width and depth to hold 1 ml of sample. Enumeration can be assessed on an average of 50 lateral and medial grids across two replicates of each sample [100]. Any protozoal cells touching the top and left lines of the grid are counted, whereas cells touching the bottom and right lines are not counted (Fig. 3b). Dilutions giving between 100 and 150 protozoa cells per 50 grids are considered optimal. Protozoa counts are expressed per millilitre of undiluted rumen fluid.

**Fig. 2.**
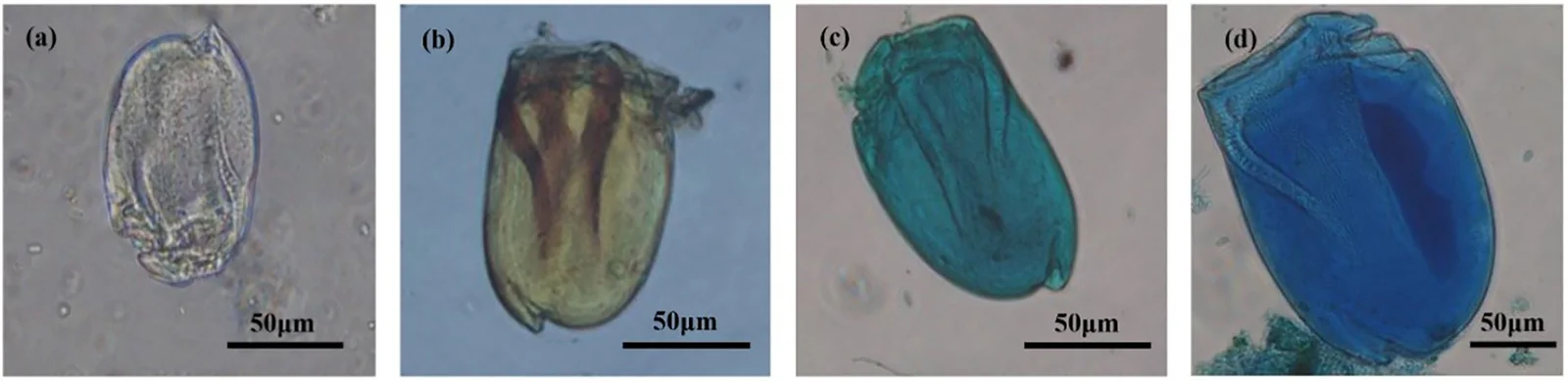
Images of *Enoploplastron triloricatum* (*Eno. triloricatum*) using different dyes. (**a**) Unstained; (**b**) Lugol’s iodine used to stain the skeletal plate of the cell; (**c**) brilliant green dye used to stain the entire cell; (**d**) methylene blue staining the macronucleus of the cell. Source – Shang *et al*. [101].

**Fig. 3.**
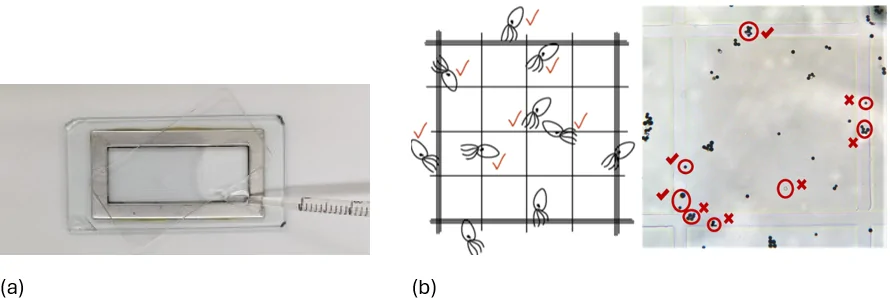
(**a**) Loading sample onto Sedgwick–Rafter counting chamber; (**b**) inclusion and exclusion of cells when counting. Source – CSIRO [147, 148].

Nuclear morphology including the size, shape and position of macronucleus or micronucleus is an important diagnostic feature of cells [106]. For differentiation between species, acidified methylene blue stains the micronucleus and macronucleus deep blue compared with the rest of the cell and can be used to identify genera and species within the Isotrichidae, Blepharocorythidae and Buetschliidae families [95, 101]. Lugol’s iodine stains the skeletal region of the cell and is useful for identifying different skeletal structures like those observed in *Diplodiniinae* [95, 101, 107]. A microscope slide with a glass coverslip is used to identify taxa at higher magnifications (450× or 1,000×). Measurements of the cell (length, width or length of nucleus, etc.) can be made using calibrated ocular scales [108].

Live protozoa can be counted; however, they are challenging to maintain viable and count due to their rapid movement over the slide. Originally used for counting sperm cells, propidium iodide, which is impermeable to intact cytoplasmic membranes, can be used to differentiate between live and dead protozoal cells, as it stains dead cells red [79, 109]. Efforts must be made to ensure protozoa remain under anaerobic conditions by flushing samples with CO_2_ throughout the procedure. Propidium iodide has also been used in combination with carboxyfluorescein diacetate, with the latter illuminating live cells green [109] and sodium thiosulfate buffer is added to rumen fluid before samples are centrifuged to pellet protozoa cells [79]. Counting is performed at 100× magnification using a fluorescence microscope fitted with a rhodamine filter to excite propidium iodide and isolate its red fluorescence for visualization [79, 109].

### Taxonomic classification using microscopy techniques

It is possible to identify most rumen protozoa to the family or subfamily level using microscopy. Protozoa can be classified further into genera or even species based on a description of distinct morphological features such as size, shape, presence or location of cilia, vacuoles and skeletal plates [108]. Genera of the subfamily Ophryoscolecinae (*Epidinium, Epiplastron, Opisthotrichum, Caloscolex* and *Ophryoscolex*) may be identified by distinctive features such as the presence or location of caudal spines or location and shape of skeletal plates [108]. However, speciation based on visual morphology is difficult, with some morphological features being stronger markers of taxonomic distinction than others [81].

In the genus *Entodinium*, *Entodinia* are the smallest and simplest species of the Entodiniomorphida protozoa, yet they are the most difficult to classify [97]. For example, the *En. caudatum* species possess three different forms of spination (ranging from none to long), depending on the presence of larger predatory protozoa [105]. *Entodinium bursa* is the largest and rarest of all Entodinia [97].

Other advanced methods of microscopy, such as FISH, are used for more detailed study of protozoa and their complex associations with other rumen prokaryotes [78, 110]. This technique is useful for exploring protozoa species diversity and symbiotic relationships within the rumen ecosystem. FISH microscopy uses oligonucleotide probes to target specific DNA or RNA sequences within cells, which are visualized under a fluorescent microscope. Specific protozoal DNA or RNA sequences can be identified, as well as intracellular bacteria and archaea, to study symbiotic relationships [78, 111–114]. The lack of ectosymbiotic methanogen–protozoa relationships in Vestibuliferida and the colonization of some Entodiniomorphida by bacteria were identified using FISH microscopy [75, 110]. A recent publication by Solomon *et al*. [114] showed using FISH that Amoebophilaceae bacteria form symbiotic relationships with members of the Entodiniomorphida order and some members of the Isotrichidae family. Current methodologies for FISH are reported by Schwarzkopf *et al*. [115] and Szulc *et al*. [116].

### Genomic resources for rumen protozoa

Robust taxonomy is crucial for scientific communication and the description of species diversity. Traditionally, taxonomic classification of rumen protozoa has largely been based on morphological observation and culture-based techniques [30, 117]. Hence, much of our conventional understanding of rumen ciliate taxonomy is constructed around indirect evidence. More recently, advances in molecular technology have permitted greater exploration of ciliate species diversity and their association with animal productivity, with a wide range of qualitative and quantitative investigations examining ciliate diversity using various molecular and microscopic techniques [7]. These approaches include restriction fragment-length polymorphism, denaturing gradient gel electrophoresis, real-time PCR (qPCR), FISH and next-generation sequencing [20, 21, 78, 118–127]. PCR technology has also allowed for the profiling of protozoa communities and quantification of protozoa within the rumen ecosystem. However, these molecular techniques provide limited insight into the genetics and metabolism of these microbes [117]. Advances in bioinformatic tools and the development of protozoal genome databases have enabled the adoption of omics technology to further enhance the study of rumen protozoa, similar to other rumen micro-organisms [117]. Genome-based taxonomy can be used to improve morphology-based classification and for the identification of new species [30]. However, 18S rRNA gene reference sequences for many protozoa genera and species are lacking. Surveys based on the 18S rRNA gene indicate that ciliate diversity is greater than morphology-based methods originally suggested [123]. The comprehensive rumen ciliate genome catalogue developed by Xie *et al.* [33] provides an important reference resource for more accurate taxonomic assignment of rumen ciliates. The resulting genomes from this study were classified into 6 families, 18 genera and 65 species, unlike earlier estimates of up to 32 genera and more than 274 species [17, 33, 108, 128]; however, the characterization of rumen protozoa still relies heavily on targeted marker genes and the availability of suitable primers. Table 1 presents the 10 different (5 forward and 5 reverse) oligonucleotide primers that were previously developed for molecular detection and characterization of protozoa.

**Table 1.** Overview of rumen protozoa primers utilized in previous studies for molecular detection and characterization

Name	Sequence (5′→3′)	Region	Amplicon length (bp)	Annealing (°C)	Reference
515FB	GTGYCAGCMGCCGCGGTAA	abV4-C	≈291	55	[2, 149]
806RB	GGACTACNVGGGTWTCTAAT	abV4-C	≈291	55	[150]
Ci28S_F	CGATAGCRWACAAGTACTRYGAA	28S	≈271	50	[140]
Ci28S_R	GACTCCTTGGTYCGTGTTTC	28S	≈271	62	[140]
Euk328f	ACCTGGTTGATCCTGCCAG	18S	≈1800	55	[151, 152]
Euk329r	TGATCCTTCYGCAGGTTCAC	18S	≈1800	55	[151, 152]
GIC1080F	GGGRAACTTACCAGGTCC	V6–V8	≈498	53.6	[78]
GIC758R	CAACTGTCTCTATKAAYCG	V6–V8	≈498	47.4	[78]
GIC1184F	TGTCTGGTTAATTCCGA	V6–V8	≈394	47.2	[78]
GIC1578R	GTGATRWGRTTTACTTRT	V6–V8	≈394	42.1	[78]
FL1_F	TCGTAACAAGGTWTCYGTAGG	ITS1	≈295	66	[140]
FL1_R	TGCWATTCGCAKTGCKTWTC	ITS1	≈295	66	[140]
FL2_F	ATGAAGAACGCAGCSAAVTG	ITS2	≈365	68.2	[140]
FL2_R	TTAAGTTCAGCGGGTRDTC	ITS2	≈365	68.2	[140]
RP841F	(AA) GAC TAG GGA TTG GAG TGG	18S	≈511 (+73)	54	[20]
Reg1302R	(TC) AAT TGC AAA GAT CTA TCC C	18S	≈511 (+73)	54	[153]
RU1_F	CACCTAGAGGAAGGAGAAGTCG	ITS1	≈200	55	[140]
RU1_R	CAYTGCATATCGCATTCTTGC	ITS1	≈200	55	[140]
RU2_F	TTCRACGATGGATGTCTTGG	ITS2	≈335	58.5	[140]
RU2_R	TGCTTAAGTCCAGCGRGTC	ITS2	≈335	58.5	[140]

### Employment of omics technology in rumen protozoa studies

The development and expansion of genome databases, coupled with advances in bioinformatic tools, offer unprecedented opportunities to investigate rumen protozoa and their role in rumen function and animal production using omics technologies (metatranscriptomics, metataxonomics, metagenomics and metaproteomics) [117]. Despite two decades of metagenomic and metatranscriptomic sequencing of the rumen microbiome, the absence of high-quality protozoal reference genomes and combined taxonomic frameworks has stunted progress on understanding rumen ciliates [129].

#### Metataxonomics

Metataxonomy was first employed in the 2010s using the 18S rRNA gene sequence [120]. This facilitated the profiling of rumen protozoa communities across a number of conditions [117]. However, metataxonomy based on the 18S rRNA gene is limited by the high conservation of this gene in eukaryotes making it difficult to discern between closely related species [23]. Preliminary molecular studies of rumen ciliates used 18S rRNA [SSU (small subunit) rDNA] sequencing to resolve the internal phylogeny of the class Litostomatea. This established the monophyletic position of rumen protozoa such as *D. ruminantium* (and related taxa) that was later expanded to include *Diplodinium dentatum*, *En. caudatum*, *Epidinium caudatum*, *Eudiplodinium maggii, I. intestinalis, Ophryoscolex purkynjei* and *P. multivesiculatum* [17, 77, 87, 130, 131]. This has since been expanded to include 450 genomes across 65 protozoa species, therefore providing a robust reference framework for the taxonomic characterization of rumen ciliates [33].

#### Genomics and transcriptomics

To date, few rumen protozoal genomes have been sequenced and made publicly available, most notably the *En. caudatum* genome [5]. Single-cell genomics approaches have enabled the recovery of genomes from individual rumen protozoa, encompassing 5 Vestibuliferida and 14 Entodiniomorphida species across 13 genera, including *Dasytricha*, *Diplodinium*, *Enoploplastron*, *Entodinium*, *Epidinium*, *Eremoplastron*, *Isotricha*, *Metadinium*, *Ophryoscolex*, *Ostracodinium* and *Polyplastron* [30]. Recent studies on protozoal genomes have revealed genes allowing for the prediction of protozoal function in the rumen ecosystem, reflecting their various ecological niches [5, 30, 32, 117]. Genomic analysis also suggested that rumen ciliates acquired many key genes via HGT from other rumen micro-organisms [5]. Single-cell sequencing and assembly-and-identification pipelines were combined to generate the first catalogue of rumen ciliate genomes that strongly correlated with previous microscopic findings [30]. This study showed that using average nucleotide identity (ANI) is a possibility for encompassing species of rumen ciliates [30]. Strong separation between genomes indicated a clear genetic difference between different protozoa species; therefore, it was possible to reclassify some species that were previously classified morphologically. For example, many *Ophryoscolecidae* have different caudal spine length and number of secondary spines, but ANI revealed that many were in fact the same species [30]. These findings were supported by 18S rRNA sequencing.

Single amplified genome genomics showed that 40,898 genes of 64,189 genes are shared between protozoa species and the remaining are unique to specific species (23,291 genes) [30]. Of the shared genes, 36% were found across both Vestibuliferida and Entodiniomorphida species with the remaining percentage confined to either order. Entodiniomorphida species were found to have larger genomes than Vestibuliferida and a larger, more diverse gene pool [30]. Genomics have also inferred functions in some species that were not previously associated with such functions. For example, it was historically thought that *Dasytricha ruminantium* was non-cellulolytic; however, genomic studies revealed that its genome encodes two cellulase genes [30]. Transcriptomic analysis has supported these genomic studies, confirming the expression of genes consistent with known functions of rumen protozoa [117]. Applying single-cell transcriptomics to individual protozoa cells gathered under different conditions has provided unique insight into ciliate metabolic dynamics [35]. The combination of genomic and transcriptomic technologies has enhanced our understanding of ciliate metabolism and function in the rumen [30].

#### Metagenomics

Metagenomics has generally been underused in rumen protozoa studies due to the lack of reference genome databases and bioinformatic tools for rumen eukaryotes [117]. Protozoa metagenomics is complicated by the complex structure of the eukaryotic genome and their relatively low abundance in the rumen ecosystem compared to their prokaryotic counterparts [117]. Metagenomic binning is usually employed to attain genomes from uncultured rumen microbes; however, this technique is not appropriate for rumen ciliates as their chromosome copy numbers are not uniform [30]. Binning requires different sequence depths among inter-genomes, but in protozoa, only contigs of similar depths can be binned into a single bin [132]. Despite this, Yan *et al*. [133] recently developed a bioinformatic software, GutEuk, to enhance the identification of eukaryotic sequences within the rumen metagenome. It can distinguish between prokaryotic and eukaryotic sequences and discern between protozoal and fungal sequences. This approach improved taxonomic and functional characterization of protozoa across more than 1,000 rumen metagenomes [133]. Targeted metagenomic approaches, such as shotgun metagenomic sequencing combined with *de novo* assembly, have also been developed to characterize the genomes of the prokaryotes associated with rumen protozoa [114]. This methodology involved sampling rumen fluid from cows, isolating the protozoa fraction, lysing the protozoa cells selectively and extracting the prokaryotic DNA. The prokaryotic DNA fraction associated with the protozoa was then sequenced and assembled, and compared with assemblies from the respective free-living prokaryotic communities, to identify genomes associated with ciliates, resulting in the recovery of 318 metagenome assembled genomes (MAGs) [114]. The development of these tools will certainly advance the study of rumen ciliates and provide further insight into protozoal influence on rumen microbiome dynamics and animal productivity [117]. Continued enhancement of binning tools will limit the challenges associated with reconstructing high-quality protozoal MAGs for identification in rumen metagenomes [117].

#### Metaproteomics

The combination of protozoa genomics, transcriptomics and metagenomics has developed a protozoa protein database enabling for metaproteomic studies of rumen ciliates [117]. Metaproteomics in complex ecosystems, like the rumen, is complicated yet essential to improve understanding of protozoa function as protein translation does not always match gene expression; hence, function must be confirmed at the protein level [134]. Metaproteomic analyses have revealed that ~50% of the rumen metaproteome can be attributed to protozoa and identified several proteins linked with multiple functions [27]. Genome-centric metaproteomics further indicates that core rumen ciliates constitute an extensive fraction of the rumen metaproteome and occupy distinct metabolic niches related to fibre and starch degradation, H_2_ production and VFA metabolism [27]. Another study identified 35,655 protein groups using metaproteomic methods, with ~18,000 of these belonging to protozoa [2]. The vast volume of protozoal proteins highlights the major functional diversity of these micro-organisms within the rumen. Hart *et al*. [134] found that the most frequent functional proteins were associated with core metabolic pathways, indicating that protein abundance reflects phylogenetic dominance. Accordingly, metaproteomes are largely dominated by protozoal genera such as *Isotricha* and *Entodinium* [2].

The application of metaproteomic studies has the potential for a more holistic understanding of rumen dynamics than sequencing alone [117, 134]. Thus, integrating metagenomic, metatranscriptomic and metaproteomic data into rumen protozoa research can help resolve the phylogenetic framework for taxonomic classification [2, 30] and enhance our understanding of the breadth of trophic niches at species level beyond our previous knowledge from biochemical and molecular findings.

### Databases used for protozoa classification

Despite being essential rumen microbes, rumen-specific protozoa reference databases were unavailable for many years. Lawther *et al*. [135] addressed this gap by developing RumenProtozoaDB v1.0.0, specifically focusing on rumen ciliates. The database was developed using 18S rRNA gene sequences from three previous studies [5, 30, 100] and contains 228 sequences (23 families, 53 genera and 100 species). RumenProtozoaDB provides sequence, taxonomy, Naive Bayes classifier and blast formats for integration into QIIME2-based workflows. Integration of the rumen ciliate genome catalogue, developed by Xie *et al*. [33], with the NCBI RefSeq database considerably improved metagenomic and metatranscriptomic read classification, increasing classification rates by an average of 11%.

The Protist Ribosomal Reference Database (PR^2^) was specifically developed to address the lack of well-curated protist SSU rRNA reference sequences, incorporating key features such as introns and organelle-derived copies commonly observed in ciliate sequences [136]. silva offers a more comprehensive, albeit less specialized, eukaryotic SSU rRNA dataset and is often used when a universal reference is needed. Nevertheless, it shares many sequences with PR^2^, enabling cross-validation between the two databases [137]. The Ribosomal Database Project (RDP) and Greengenes databases, although not specifically designed for protist studies, contain a limited number of curated eukaryotic entries and may therefore be useful for the preliminary taxonomic screening of ciliate amplicon data [138]. Ultimately, all curated databases rely on sequences deposited in the NCBI GenBank repository, which remains the authoritative source for newly submitted ciliate SSU rRNA sequences [139]. Somasundaram *et al*. [140] developed an in-house rRNA operon database (18S–ITS1–5.8S–ITS2–28S) for rumen ciliate taxa due to the limited availability and incompleteness of publicly available databases. This database is referred to as the Rumen Ciliate rRNA Operon Database (RCROD) and was developed using the RESCRIPt plugin in QIIME2 (version 2024.2). It incorporates 52 previously reported single-cell amplified genomes of rumen ciliates. As the silva and PR2 databases only contain a few rumen ciliate sequences, the RCROD database fills taxonomic gaps for rumen-associated taxa. Table 2 presents the major reference databases for protozoa 18S rRNA classification.

**Table 2.** Major reference databases for rRNA-based taxonomic classification of protozoa (ciliates)

Database	Main features for protozoa	Typical application
**Rumen Ciliate Genome Catalogue**	A rumen-specific catalogue comprising 450 ciliate genomes (59 previously published datasets), with 87% newly sequenced The catalogue represents 65 species across 18 genera and provides a high-resolution genomic framework for rumen ciliates [33]	Genome-based taxonomy, high-resolution community profiling Increases metagenomic read classification by ~11% over RefSeq alone [33]
**RumenProtozoaDB**	To improve the taxonomic characterization of rumen protozoa, a curated 18S rRNA reference database has recently been established, comprising 100 species of rumen ciliates Built from publicly available datasets, including 168 sequences, 65 single-amplified genome assemblies and the *En. caudatum* macronuclear genome [5, 30, 100]; 18S rRNA genes were extracted using Barrnap v0.9, and redundant sequences were removed by CD-HIT clustering at 100% sequence identity and length	Integration into metataxonomic workflows such as QIIME2 for reliable taxonomic assignment of rumen protozoal sequences Direct use in QIIME2 through the pre-trained Naive Bayes classifier or pre-formatted blast database, without additional classifier training Improved taxonomic resolution of rumen protozoal communities in diversity surveys
**RCROD**	Complete rRNA operon (18S-ITS1-5.8S-ITS2-28S) sequences from 20 rumen protozoa species; universal ITS1/ITS2 and 28S D1-D2 primers; ITS1 region has highest sequence variability, enabling species-level resolution [140]	Species-level metataxonomic profiling of rumen ciliates; nutrition and microbial ecology studies
**PR^2^**	Curated collection of SSU rRNA (18S) sequences for unicellular eukaryotes (protists), including ciliates Provides nuclear-encoded sequences, as well as organelle copies (mitochondrial, plastid, and hydrogenosomal), with intron handling and removal of chimaeras Uses an eight-rank taxonomy system that facilitates the automated classification of ciliate sequences	Taxonomic assignment of environmental 18S amplicons from rumen or other gut samples Designing primers and evaluating primer coverage for protozoan surveys Comparative analyses of ciliate diversity across habitats
**silva**	Large SSU rRNA reference set that includes eukaryotic sequences; a subset of the silva release contains curated protist entries Uses the NCBI taxonomy and applies strict length/quality filters	Broad phylogenetic placement of ciliate sequences when a universal SSU reference is needed Useful for building phylogenetic trees that combine bacterial, archaeal and eukaryotic lineages
**RDP**	Primarily a prokaryotic SSU rRNA resource but also offers a limited set of curated eukaryotic sequences	Quick taxonomic checks of ciliate 18S reads when only a few protist sequences are present
**Greengenes**	Like RDP, focused on bacterial 16S rRNA, with a small collection of eukaryotic entries	Occasionally used as a fallback reference for mixed-kingdom amplicon datasets
**NCBI GenBank**	The primary repository for all publicly deposited ciliate SSU rRNA sequences; not curated for taxonomy but serves as the source data for the above specialized databases	Retrieval of specific ciliate gene sequences, reference sequence verification and manual curation

## Current knowledge gaps

A comprehensive understanding of the rumen microbiome is fundamental to enhance livestock productivity and sustainability. However, reconstruction of the rumen microbiome has largely focused on the bacterial and archaeal populations, with the protozoal community long overlooked [27]. This is driven by the challenges associated with culturing rumen eukaryotes in an aseptic environment, and the complexity of their genomes hindering metagenomic assembly and binning approaches. Modern sequencing and molecular techniques are imperative to truly reveal the role of protozoa in the rumen and help influence the rumen microbiome to improve livestock efficiency. Recent advances in sequencing and database development have substantially improved the resources available for rumen protozoa. The rumen ciliate genome catalogue developed by Xie *et al.* [33], and the RumenProtozoaDB reference database established by Lawther *et al.* [135], provide comprehensive genomic reference frameworks for rumen ciliates. Nevertheless, continued expansion of reference genomes is required to further resolve rumen protozoal diversity and improve comparative analyses and taxonomic assignments in the rumen ecosystem. While both of these resources are extensive, they are limited by the lack of publicly available data for rumen ciliates and are confined to 18S rRNA regions, excluding alternative rRNA regions (ITS or 28S), which could also be used for protozoa classification [135].

The influence of specific protozoal taxa on rumen dynamics remains ambiguous as studying individual species is highly dependent on their cultivability [19, 23, 141]. There has yet to be any significant breakthrough in achieving long-term, large-scale cultivations of rumen protozoa. Therefore, microbiologists rely on monoaxenic cultures of ciliate protozoa growing in the presence of bacteria, yet it is difficult to distinguish the functions of protozoa from those of bacteria [55]. Protozoal cultures are extremely difficult to establish as morphologically similar cells are used for inoculation; however, many species share similar morphology, increasing the likelihood of inoculating more than one species. Even after successfully establishing cultures, several protozoa cells will die after a certain number of cell divisions [15]. While there has been some success in culturing species of Entodiniomorphida, establishing long-term cultures of Vestibuliferida species remains challenging [23, 142]. Currently, most studies investigating specific species of rumen protozoa have inoculated a single species into defaunated ruminants to achieve a mono-faunated status [15]. However, mono-faunation is only assumed as morphologically similar cells could represent different species [15]. Additionally, the maintenance of mono-faunated animals is difficult and impractical [23]. Obtaining axenic cultures of protozoa would enable more detailed morphological and molecular characterization of these micro-organisms and provide insight into the interactions of specific taxa with other rumen micro-organisms. This would be beneficial for microbiome research and enhance understanding of rumen microbial community dynamics.

As protozoa are difficult to maintain and yet such a crucial part of the rumen microbiome, optimizing preservation techniques to increase their survival rates and cell viability would be beneficial. Bulk storage of rumen fluid would offer researchers a quality alternative source of rumen inoculum compared to the maintenance of fistulated donors or harvesting from euthanized donors. Cryopreservation of rumen digesta offers potential to store rumen digesta for use in *in vitro* studies, limiting the need for maintaining fistulated animals and therefore reducing the number of surgeries to be carried out on cattle or sheep. Storing rumen fluid whilst maintaining its microbial activity would also enable standardization between *in vitro* studies [143]. Cryoprotectants have been used to protect cell membranes by limiting cell dehydration during freezing [144]. Dimethyl sulfoxide has been identified as an effective preservation method compared to preservation with glycerol [145]. Tunkala *et al.* [146] reported a significant decline in the numbers of protozoa following cryopreservation but the technique is still deemed suitable for *in vitro* experiments as a ranking tool for diets. Preserved rumen fluid could be evaluated using microscopic techniques mentioned in the present review, particularly aimed at the counting of live cells to assess protozoa viability.

The time and expertise required for taxonomic identification have limited microscopy-based approaches and contributed to its decline in recent decades especially with molecular-based techniques widely available. Whilst some studies investigate the integration of artificial intelligence software into the counting procedure to improve the speed and efficacy of microscopy, further work is needed to improve the accuracy and reliability of trained models [101]. Whilst microscopy enumeration remains the gold standard, only a few skilled researchers can accurately identify species by morphology, one of whom was Dehority, who massively contributed to this area of research in past decades [81]. Morphology-based identification is also highly laborious and must be combined with genomics for accuracy. A modernization of microscopic enumeration techniques through integration with artificial intelligence could renew interest in these techniques to the next generation of researchers. Many studies assessing the use of anti-methanogenic supplements in the diets of live cattle often incorporate amplicon sequencing measures [14]. Perhaps there is scope to introduce the counting of protozoa into live animal studies in combination with molecular-based techniques to help corroborate results.

## Conclusion

This literature review provides insight into the structure, functions, and importance of rumen protozoa; describes key methodologies for their study and identification including classical microscopy techniques, genomic technologies and reference databases available; and highlights remaining knowledge gaps. Rumen protozoa are an important yet understudied part of the rumen ecosystem. Despite interest in their ecological roles having grown in recent years, progress is still hindered by long-standing methodological limitations. Classical microscopy remains the most reliable method of identification and enumeration but it requires specialist expertise and is difficult to scale up. AI-assisted image analysis is an emerging tool showing promise in reforming these techniques; however, further refinement and validation are required before it can be used for routine protozoal study. Current protocols do not fully preserve protozoal vitality and the long-term effects on the microbial community are unclear. More robust preservation methods are required to ensure consistent protozoal activity in experimental systems.

The most significant limitation to the study of rumen protozoa is the difficulty in obtaining pure or axenic rumen protozoa cultures. The dependency of protozoa on bacteria, morphological similarity between species and the challenges involved in maintaining long-term viability have all limited both cultivation and downstream functional studies. These barriers also directly impact molecular research, as the scarcity of validated reference sequences, particularly for the main rumen taxa, Vestibuliferida and Entodiniomorphida, results in insufficient databases. Consequently, taxonomic resolution in amplicon and metagenomic studies remains inadequate.

In summary, while the importance of rumen protozoa is increasingly recognized, substantial gaps in culturing, taxonomy and database representation still hinder progress. Advancing the study of rumen protozoa will require coordinated efforts to improve culturing techniques, expand reference databases, refine cryopreservation strategies and integrate computer-assisted technology with classical microscopy. Strengthening these foundations is essential for achieving a more accurate and comprehensive understanding of protozoal diversity and function within the rumen and continuing efforts to improve livestock efficiency, productivity and sustainability.

## Abbreviations

ANI: average nucleotide identity
CAZyme: carbohydrate active enzyme
GH: glycoside hydrolase
HGT: horizontal gene transfer
MAG: metagenome assembled genome
PR^2^: Protist Ribosomal Reference Database
RCROD: Rumen Ciliate rRNA Operon Database
RDP: Ribosomal Database Project
SSU: small subunit
VFA: volatile fatty acid
